# Management of Satellite Lesions in Hepatocellular Carcinoma: An Updated Review

**DOI:** 10.1002/jso.70298

**Published:** 2026-06-01

**Authors:** Andy Tran, Mohammed O. Suraju, Ihuoma Tasie, Abdelrahman K. Abdalla, Hassan Aziz

**Affiliations:** ^1^ Department of Surgery University of Iowa Health Care Iowa City Iowa USA; ^2^ Meharry Medical College Nashville Tennessee USA

**Keywords:** imaging, liver, metastasis, outcomes, satellite

## Abstract

Satellite lesions, defined as microscopic or small macroscopic tumor nodules within 2 cm of a primary hepatocellular carcinoma (HCC), represent early intrahepatic dissemination and are strongly associated with aggressive tumor biology. This review synthesizes the current evidence regarding the biological basis, diagnostic challenges, prognostic relevance, and therapeutic approaches for managing satellite lesions in HCC. In published studies, satellite lesions were consistently linked to aggressive tumor behavior, including higher rates of microvascular invasion, multifocality, and early postoperative recurrence. Patients with satellite lesions experienced significantly reduced disease‐free and overall survival compared to those with solitary tumors, although outcomes varied with tumor size, number, and liver function. In transplant‐eligible cohorts, satellite lesions were associated with exceeding standard listing criteria and demonstrated increased post‐transplant recurrence when identified on explant pathology. Radiologic detection showed moderate sensitivity but high specificity, influencing selection for resection, ablation, and transplant‐based strategies. Satellite lesions are a critical marker of tumor aggressiveness in HCC and significantly influence surgical and transplant decision‐making. Although resection may be appropriate for carefully selected patients with preserved liver function, recurrence rates remain high. The presence or suspicion of satellite lesions strongly impacts transplant candidacy, with most guidelines considering radiological satellites an indicator of advanced disease.

## 1 Introduction

Hepatocellular carcinoma (HCC) is the third leading cause of cancer‐related death worldwide, with a 5‐year survival rate of approximately 18% [1]. A significant proportion of patients present with multiple intrahepatic tumors, which have been associated with poorer outcomes [2]. One category of secondary nodules in HCC is satellite nodules, which have been defined as tumors < 2 cm in size, located < 2 cm from the primary tumor [3, 4]. These nodules may be occult and are often first identified in patients who present with an apparently solitary tumor on pathology [5]. These lesions represent intrahepatic metastasis from the same primary cancer as opposed to multicentric HCC, which arises as a separate primary tumor [4]. Their presence signals a biologically aggressive disease and contributes to high rates of recurrence following curative‐intent therapy [6].

Given the increasing use of surveillance imaging and the growing population of patients considered for resection, transplantation, or locoregional therapy, clinicians must understand the implications of satellite lesions and their influence on treatment pathways. This review summarizes the current knowledge on the pathophysiology, imaging characteristics, prognostic impact, and management of satellite lesions in HCC.

## 2 Methods

This narrative review was conducted to contextualize the current body of knowledge regarding the clinical management of satellite lesions in HCC. PubMed and Google Scholar were queried for studies published between 2000 and 2025 using combinations of the following keywords: “hepatocellular carcinoma,” “satellite lesion,” “intrahepatic metastasis,” “microvascular invasion,” “treatment,” “diagnosis,” and “liver transplantation.” Original studies, systematic reviews, and clinical practice guidelines were included. Priority was given to studies specifically evaluating satellite lesions and related biological features, including microvascular invasion and intrahepatic spread, while broader HCC literature was selectively incorporated to provide a clinical context where satellite‐specific data were limited. The reference lists of selected articles were also screened for additional relevant studies. As such, several included references fall outside of the aforementioned publication timeframe. As a narrative review, this study is inherently subject to selection bias; however, efforts were made to provide a balanced and clinically relevant synthesis of the available evidence.

### 2.1 Pathophysiology

Satellite nodules are small tumor lesions located close to the primary tumor and are considered a form of intrahepatic metastasis. Disease progression follows the sequence of capsular invasion to extracapsular invasion, vascular invasion primarily via the portal venous system, and intrahepatic metastasis [7, 8, 9]. These nodules are closely associated with microvascular invasion, further reinforcing their significance in metastasis [10]. Satellite nodules are contrasted with multicentric carcinogenesis, which is characterized by genetically unrelated tumors arising at separate locations in a chronically diseased liver [4].

The definition of satellite lesions varies across the literature and is most commonly described as nodules ≤ 2 cm located within 2 cm of the primary tumor; however, this definition is largely based on histopathologic assessment and is not uniformly applied [7]. In practice, there is considerable conceptual overlap between satellite nodules and intrahepatic metastases, as both represent tumor spread from the primary lesion and share similar biological behavior. In contrast, multicentric HCC arises from independent tumor clones within a chronically diseased liver and carries distinct prognostic implications [8]. Importantly, these distinctions are often difficult to establish preoperatively, as radiologic imaging frequently underestimates the presence of small satellite lesions and cannot reliably differentiate between these entities. As such, discrepancies between radiological and pathological definitions limit the clinical applicability of strict size‐ and distance‐based criteria. However, to allow for the integration of satellite lesions into clinical decision‐making frameworks, a standardized clinical definition must be established. Based on the available evidence, defining a satellite lesion as a nodule ≤ 2 cm located within 2 cm of the primary tumor *within the same segment* will accurately identify a large majority of these nodules.

### 2.2 Incidence

The reported prevalence of satellite lesions appears to be highly variable in literature. Various studies have reported rates ranging from 15% to 42%, depending on the population and disease burden [6, 11, 12, 13]. It is common to find satellite lesions in patients with small primary tumors. Previous studies have reported the presence of satellite lesions in HCC less than or equal to 3 cm between 19% and 38% [11, 13, 14].

### 2.3 Diagnostic Considerations

#### 2.3.1 Imaging

Advances in multiphasic CT and MRI have improved the detection of small intrahepatic lesions; however, identifying satellite nodules remains challenging [15]. This limitation is primarily due to the small size of satellite lesions and their frequent subcentimeter distribution within the surrounding liver parenchyma, often placing them below the spatial resolution of conventional imaging techniques [16, 17, 18, 19]. This is reinforced by the fact that LI‐RADS diagnostic criteria have not been proposed for nodules < 1 cm [20]. MRI with hepatobiliary contrast has the highest sensitivity, particularly for lesions smaller than 1 cm [21]. Typical imaging features include arterial phase hyperenhancement, washout in the portal venous or delayed phases, and hypointensity in the hepatobiliary phase. While these characteristics form the basis for HCC diagnosis on imaging, satellite nodules often fail to demonstrate sufficiently distinct enhancement patterns to meet the formal radiographic thresholds [16, 21, 22]. Accordingly, intraoperative ultrasound should be considered an essential adjunct during hepatic resection and should be performed systematically to prevent missing any lesions [23].

Several imaging features are associated with an increased likelihood of satellite or microvascular invasion. These include peritumoral arterial enhancement, nonsmooth tumor margins, peritumoral hepatobiliary phase hyperintensity, and hepatic or portal vein compression, which have been described as radiological biomarkers of aggressive tumor biology [24, 25]. These findings likely reflect microscopic tumor infiltration into the surrounding liver tissue rather than discrete radiologically visible lesions [26]. Although satellite nodules may not be visualized, such biomarkers may aid in preoperative risk stratification and treatment selection [15].

#### 2.3.2 Pathology

Histopathological evaluation remains the definitive method for diagnosing satellite lesions. The reported prevalence varies widely depending on tumor size and selection criteria, ranging from approximately 10% in small solitary HCCs to more than 50% in larger tumors [24]. This variability reflects both differences in tumor biology and the extent of histologic sampling, as satellite lesions are often microscopic and may be missed without thorough examination of adjacent liver tissue. Satellite lesions are distinguished from multicentric tumors by their high degree of shared genetic mutations [27]. Pathological detection of satellite lesions frequently coincides with microvascular invasion and poor differentiation, confirming their role as a histological surrogate of aggressive tumor biology [24]. These features also suggested microscopic metastasis beyond the dominant lesion [28]. From a histopathological standpoint, satellite nodules are, therefore, not merely secondary tumor foci, but rather a manifestation of biologically aggressive disease with increased metastatic potential.

Given the strong association between satellite lesions, microvascular invasion, and poor differentiation, meticulous pathological assessment following resection or transplantation is critical. Identification of satellite nodules on histologic examination provides prognostic information that is not reliably captured by preoperative imaging alone, particularly in tumors that otherwise meet the criteria for early‐stage disease [29]. Therefore, pathology plays a central role in accurately characterizing tumor aggressiveness and informing postoperative risk stratification.

#### 2.3.3 Prognostic Implications

Patients with satellites have significantly higher rates of early recurrence after resection, often occurring within the first 2 years [30]. This pattern of early recurrence is widely interpreted to reflect pre‐existing microscopic intrahepatic metastases rather than de novo tumorigenesis, suggesting that satellite lesions represent established tumor dissemination at the time of treatment [31, 32]. Satellite lesions also independently predict reduced overall survival and disease‐free survival, reinforcing their role as markers of aggressive tumor biology that adversely affects long‐term outcomes regardless of the initial treatment intent [6, 28, 32, 33].

In the transplant setting, the identification of satellite lesions on explants is associated with higher post‐transplant recurrence, particularly when accompanied by microvascular invasion or poor differentiation [34]. These findings underscore the importance of careful pathologic assessment of explanted livers, as satellite lesions may identify patients at higher risk of recurrence despite meeting the conventional transplant eligibility criteria [34]. Although transplantation still offers favorable long‐term outcomes for selected patients, satellite lesions portend a higher risk profile and may warrant intensified post‐transplant surveillance or consideration of adjunctive strategies [35].

Notably, the prognostic significance of satellites persists across tumor sizes and stages. Even in tumors ≤ 5 cm in size, the presence of satellites can shift the expected survival toward those with more advanced disease [29]. Collectively, these findings support the incorporation of satellite lesion status into postoperative risk stratification and underscore its relevance in guiding surveillance intensity and adjuvant treatment.

### 2.4 Management Strategies

Treatment strategies for HCC are thoroughly outlined in several widely utilized national and institutional guidelines that incorporate clinicopathological characteristics, multidisciplinary reviews, and multimodal treatment. Treatment strategies are broadly categorized as curative‐intent surgical intervention for early‐stage disease, locoregional control for intermediate‐stage disease, and systemic therapy for advanced disease. Clinical decision‐making is dictated by patient performance status, tumor burden, and liver function, assessed using the Child‐Turcotte‐Pugh score or Model for End‐Stage Liver Disease (MELD) score [36].

The NCCN HCC guidelines do not include a discrete recommendation or unique pathway specifically for “satellite lesions” as a separate entity in decision algorithms. Satellite nodules were not named distinctly in the diagnostic or treatment flowchart. Instead, the broader concept of additional intrahepatic nodules or multifocal disease within the context of staging, transplant criteria, and locoregional systemic planning were discussed (Table 1).

**Table 1:** Societal guidelines and recommendations regarding satellite nodules.

Guideline	Explicit discussion of satellite nodules	Recommendations regarding satellite nodules	Recommendations independent of multinodular disease
NCCN [20]	Yes	Management is dictated based on how satellites affect overall tumor burden, staging, resectability.	No
EASL [37]	Yes	Satellitosis is treated as a high‐risk feature rather than multifocal disease.	Yes
		– Liver resection is recommended in noncirrhotic livers with single HCC regardless of the presence of satellite lesions whereas multifocal disease involving multiple segments should receive downstaging treatment prior to surgical evaluation.	
		– If pathologically identified following resection, the presence of satellites or other high‐risk features may be considered for pre‐emptive liver transplantation.	
BCLC [38]	Yes	The presence of satellite nodules is predictive of recurrence and may support pre‐emptive liver transplantation following resection.	Yes
AASLD [35]	Yes	Satellite nodules are a high‐risk feature that increases the risk of recurrence and should be considered when determining the need for adjuvant therapy and post‐resection surveillance, though explicit recommendations are not provided.	Yes Satellite

### 2.5 Surgery

Surgical resection is considered the foundational curative treatment for HCC. According to these guidelines, the ideal candidate for surgical resection is a patient with appropriate liver function (Childs‐Turcotte‐Pugh Class A cirrhosis or no cirrhosis) without clinically significant portal hypertension bearing a single lesion without major vascular invasion, although select patients with multifocal disease may also be considered [20, 35, 37, 38].

When upfront surgery is selected, anatomical resection should generally be favored over nonanatomical resection when satellite lesions are suspected [39]. This is because satellite lesions are markers for intrahepatic metastasis, are associated with aggressive tumor biology, and confer a high risk of recurrence if left behind [40, 41, 42]. Anatomic resection removes the entire tumor‐bearing portal territory and may capture microscopic spread along the portal venous tributaries. This accounts for the primary mechanism of HCC dissemination, making it a more effective oncologic approach, as it more reliably facilitates negative margin resection [43]. Notwithstanding, non‐anatomical resection may still be considered for small, peripherally located HCCs, particularly in patients with limited hepatic reserve [39]. However, it should be noted that satellite nodules are associated with an increased risk of recurrence even among small lesions [44].

Importantly, the decision to resect and the extent of resection should consider patient‐specific factors, including liver function assessment (Child‐Pugh class, MELD score), severity of portal hypertension, and other comorbidities that may affect perioperative risk and long‐term survival (e.g., cardiac and pulmonary diseases) [39, 45]. Even with optimal surgical techniques, recurrence rates following resection remain substantially high (approximately 50%–70% at 5 years), highlighting the importance of thoughtful surgical selection [35]. When possible, patients should be discussed on a multidisciplinary board and performed by experienced surgeons at high‐volume centers [46, 47].

### 2.6 Liver Transplantation

Liver transplantation offers the most comprehensive oncologic approach by removing both the tumor‐bearing liver and the diseased hepatic parenchyma [48]. Historically, the Milan criteria, which were created to select candidates with the lowest recurrence risk, excluded patients with multiple nodules beyond the “single lesion ≤ 5 cm or up to three lesions each ≤ 3 cm” framework [49]. Given that satellite lesions often appear as small additional nodules, their presence on imaging commonly places a patient outside the Milan criteria and, therefore, outside standard listing eligibility [36]. However, this is a staging limitation, rather than an absolute biological contraindication. Patients with satellite lesions identified only on explant pathology still achieve acceptable survival, although recurrence rates are higher than those in patients with truly solitary tumors [35, 50]. Therefore, satellite nodules are best viewed as high‐risk pathologic features rather than exclusionary criteria. Patients within the Milan criteria but suspected of having satellites remain eligible for transplantation, while downstaging with response‐based selection is increasingly employed for those initially outside the Milan criteria, as the integration of locoregional treatments prior to transplantation may mitigate some of the aggressive biological behavior associated with satellites [51, 52].

Liver transplantation may also be utilized as salvage therapy following recurrence after surgical resection. Traditionally, salvage liver transplantation was not implemented until patients demonstrated disease recurrence. More recently, however, liver transplantation following surgical resection has been proposed in patients whose disease demonstrates high‐risk features even before disease recurrence, a process known as ab initio transplantation. Satellite nodules and microvascular invasion are the two most frequently cited risk factors prompting ab initio transplantation. The rationale behind this is that the presence of these risk factors predicts not only recurrence, but also recurrence beyond Milan criteria, leaving these patients with no possible curative option [6, 53, 54, 55]. The counterargument to this being that the presence of these risk factors may also predict disease so aggressive such that the risk for post‐transplantation recurrence is similarly elevated and these patients likely would have failed primary liver transplantation as well, as is noted by the AASLD [35]. Though there are no studies to our knowledge directly comparing ab initio transplantation versus primary transplantation in the context of satellite nodules, data from Ferrer‐Fàbrega and colleagues suggest good outcomes in patients undergoing ab initio transplantation follow surgical resection with 5‐year survival 82.4% [55].

The adoption of ab initio transplantation following resection must be balanced not only by clinical indication, but also by the chief challenge in transplant medicine, namely, organ scarcity and subsequent prolonged wait times [56]. Many patients ultimately drop off the transplant waitlist because of disease progression. To address this limitation, living donor liver transplantation has emerged as an alternative strategy to expand the donor pool and reduce waitlist [36]. Regardless of the approach, vigilant post‐transplant surveillance is essential as recurrence remains a leading cause of mortality [48].

### 2.7 Ablation

While treatment guidelines do not directly address satellite lesions in the context of ablation, ablation is recommended as the preferred locoregional therapy for potentially resectable or transplantable patients [20], in patients who are not candidates for transplantation [35, 38], or in those who are not candidates for or decline surgical resection [35]. However, multinodularity is an independent risk factor for reduced recurrence‐free and overall survival when treated with ablation [57, 58]. Recent studies have suggested that liver resection is superior to nonsurgical treatment for multinodular disease [59, 60].

Therefore, the use of thermal ablation, including radiofrequency and microwave ablation, should be reserved for patients who are not optimal surgical candidates or undergo surgery. Tumor size is a critical determinant of efficacy because larger tumors are more likely to harbor satellite lesions beyond the ablation zone [61, 62]. Ablation is the most effective treatment for tumors ≤ 3 cm in diameter [63]. For lesions > 3 cm, combination approaches such as ablation with transarterial chemoembolization (TACE) or limited resection should be considered when feasible [22, 62, 64]. The location of lesions near major vessels, bile ducts, or other organs will additionally limit the effectiveness or pose an undue risk of this modality [20, 65]. The use of multiple electrodes or creation of overlapping ablation zones with a single electrode should be considered in patients with or at risk of satellite nodules to achieve ablative margins of at least 1 cm [61]. Ablation may also be utilized as a bridge to transplantation to halt disease progression or downstaging in the interim [20, 35, 37].

### 2.8 Intra‐Arterial Therapy

TACE and trans‐arterial radioembolization (TARE) are two intra‐arterial modalities that exploit the arterial supply of HCC tumors while sparing the normal liver parenchyma, which still receives blood through the portal circulation [35, 36, 66]. Intra‐arterial chemotherapy injection with TACE is followed by embolization for arterial occlusion, while TARE delivers β‐emitting yttrium‐90 (Y90) microspheres to induce tumor necrosis [36]. Among NCCN, BCLC, and AASLD, TACE is recommended as the first‐line treatment for BCLC stage B disease, although TARE is an appropriate alternative [20, 35, 38]. Patient selection is critical because advanced liver dysfunction, portal vein thrombosis, and high tumor burden may preclude these therapies [35, 66]. Data on comparative effectiveness are largely mixed [35, 67, 68, 69]; however, TARE may be associated with a longer time to progression [70]. As such, the choice of intra‐arterial therapy is largely dependent on the center's expertise, availability, and patient factors. Combination strategies such as TACE followed by ablation have demonstrated excellent outcomes in high‐risk tumors and may be beneficial for patients with lesions at risk of harboring satellite lesions [64, 71]. The AASLD guidelines note that radiation segmentectomy, a form of TARE that administers an ablative dose of Y90 to one or two angiographic liver segments, may be effective in treating microsatellites [35]. Additional roles for intra‐arterial therapies include early or very early disease in patients for whom surgery is not an option or a bridge to transplantation.

### 2.9 Systemic Therapy

Systemic therapy has undergone substantial evolution, particularly with the advent of targeted agents and immunotherapies. Sorafenib was the first systemic therapy that showed improved survival rates; however, multiple other therapies, including immune checkpoint inhibitors, have been discovered since then [35, 66]. Regimens, such as atezolizumab with bevacizumab or durvalumab‐based combinations, have become first‐line options for advanced HCC and are being investigated in neoadjuvant and adjuvant settings. In patients with contraindications to these regimens, sorafenib or lenvatinib remain an appropriate alternatives [35]. Cabozantinib, regorafenib, ramucirumab, and pembrolizumab are considered second‐line therapies, but have limited evidential support. Emerging data suggest that patients with high‐risk pathological features, including satellite lesions, may benefit from adjuvant systemic therapy; however, definitive recommendations await ongoing trial results [35]. Some recent studies evaluating the potential benefits of neoadjuvant immunotherapy in HCC have also shown promise.

### 2.10 Guideline Recommendations

**Figure 1:**
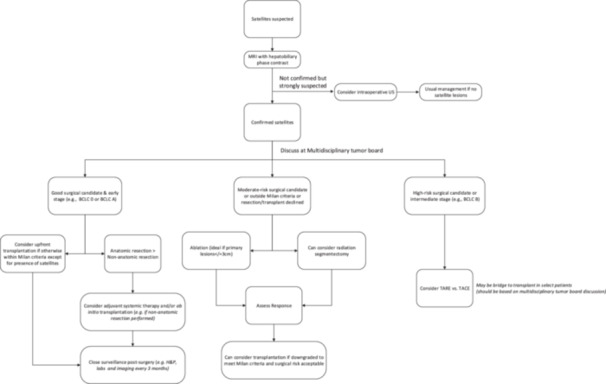
Proposed algorithm for working up patients with satellite nodules.

There is little mention of satellite lesions in the most utilized guidelines (AASLD, EASL, BCLC, and NCCN). Satellitosis is recognized as a marker of aggressive tumor biology but does not significantly contribute to clinical decision‐making outside of staging implications their identification may confer. EASL guidelines provide the most direct recommendation for managing satellite nodules with the recommendation that surgical resection be the preferred treatment for solitary HCC *with or without satellite nodules* in a noncirrhotic liver. Both EASL and AASLD comment that radiation segmentectomy may be a feasible treatment strategy for managing both primary and associated satellite tumors [35, 37]. Beyond these recommendations, there are no recommendations that independently address satellite nodules. EASL and BCLC, both regarding the presence of satellite nodules in the context of high‐risk features, indicate their presence may support pre‐emptive transplantation prior to disease recurrence following resection [37, 38]. In this setting, satellite lesions are discussed in the context of high‐risk pathologic features or multifocal disease as a singular entity. Satellite nodules are high‐risk features associated with poor outcomes. The dearth of recommendations regarding clinical management in this context is disproportionate to its clinical impact (Figure 1).

### 2.11 Future Directions

Despite the increasing recognition of satellite lesions as an indicator of aggressive tumor biology in HCC, their optimal detection and management remain incomplete. Although significant advancements in the characterization and risk stratification of satellite lesions in HCC have occurred in recent years, there remains an unmet need for improved preoperative detection and risk prediction. Current and future research focused on improving the preoperative detection of satellite lesions through advanced imaging technologies, radiomics, and machine learning models has demonstrated significant potential. In conjunction with the use of novel blood‐based biomarkers, these emerging technologies may help to better characterize patients with high‐risk diseases to individualize treatment strategies. Therefore, further research exploring the role of locoregional and systemic therapies in the treatment of satellite lesions is warranted. Ultimately, satellite lesions should be evaluated not only as a morphologic finding but as a dynamic biomarker of tumor biology that can be integrated into future risk‐adapted treatment algorithms.

## 3 Conclusion

Satellite lesions are a critical determinant of prognosis in HCC and reflect aggressive tumor biology characterized by early intrahepatic dissemination. When identified on imaging, they may significantly influence the treatment options; however, many satellite nodules remain occult until pathological examination. Emerging advances in imaging, biomarkers, and systemic therapies hold promise in refining risk stratification and improving outcomes. Multidisciplinary individualized treatment approaches remain essential for the effective management of patients with suspected or confirmed satellite lesions.

## Funding

The authors have nothing to report.

## Disclosure

The authors have nothing to report.

## Ethics Statement

All procedures performed in this study involving human participants were in accordance with the ethical standards of the institutional and/or national research committee and the 1964 Helsinki Declaration and its later amendments or comparable ethical standards.

## Conflicts of Interest

The authors declare no conflicts of interest.

## SYNOPSIS

Satellite lesions in hepatocellular carcinoma are associated with significantly worse disease‐free and overall survival compared to solitary tumors, reflecting aggressive tumor biology and early intrahepatic dissemination. Their presence is variably influenced by tumor size, number, and underlying liver function. Although carefully selected patients with preserved hepatic reserve may still undergo resection, recurrence rates remain high, underscoring the biologic significance of satellitosis. In the transplant setting, suspected or occult satellite lesions strongly impact patient selection and risk stratification, as they are widely regarded as indicators of advanced disease. Collectively, satellite lesions play a central role in guiding surgical and transplant decision‐making despite ongoing variability in their preoperative detection.

## Data Availability

The data that support the findings of this study are available on request from the corresponding author. The data are not publicly available due to privacy or ethical restrictions.
